# Navigating Design Options for Large-Scale Interprofessional Continuing Palliative Care Education: Pallium Canada's Experience

**DOI:** 10.1089/pmr.2021.0023

**Published:** 2021-08-13

**Authors:** José Pereira, Gordon Giddings, Robert Sauls, Ingrid Harle, Elisabeth Antifeau, Jonathan Faulkner

**Affiliations:** ^1^ Pallium Canada, Ottawa, Ontario, Canada.; ^2^ Division of Palliative Care, Department of Family Medicine, McMaster University, Hamilton, Ontario, Canada.; ^3^ Institute of Culture and Society (ICS), University of Navarra, Navarra, Spain.; ^4^ College of Physicians and Surgeons of Alberta, Edmonton, Alberta, Canada.; ^5^ Mississauga, Ontario (retired), Canada.; ^6^ Palliative Care End of Life Services, Interior Health, Vancouver, British Columbia, Canada.

**Keywords:** continuing professional development, education, instructional design, interprofessional, palliative care

## Abstract

To be effective, palliative care education interventions need to be informed, among others, by evidence and best practices related to curriculum development and design. Designing palliative care continuing professional development (CPD) courses for large-scale, national deployment requires decisions about various design elements, including competencies and learning objectives to be addressed, overall learning approaches, content, and courseware material. Designing for interprofessional education (IPE) adds additional design complexity. Several design elements present themselves in the form of polarities, resulting in educators having to make choices or compromises between the various options. This article describes the learning design decisions that underpin Pallium Canada's interprofessional Learning Essential Approaches to Palliative Care (LEAP) courses. Social constructivism provides a foundational starting point for LEAP course design, as it lends itself well to both CPD and IPE. We then explore design polarities that apply to the LEAP courseware development. These include, among others, which professions to target and how to best support interprofessional learning, class sizes, course length and content volume, courseware flexibility, regional adaptations, facilitator criteria, and learning methods. In some cases, compromises have had to be made between optimal perfect design and pragmatism.

## Introduction

The need for palliative care education for health professionals is recognized.^1–4^ This applies across professions, care settings, and the learning continuum, from undergraduate and postgraduate training to continuing professional development (CPD). To be effective, these education interventions need to be informed, among others, by evidence and best practices related to curriculum development and design.^5–9^

Although there are many definitions for *curriculum*, *curriculum development*, and *curriculum design*, they broadly include the competencies to be acquired, learning objectives, teaching and learning strategies, delivery methods, course content and materials, learner assessment, and program evaluation approaches.^10^ They also encompass planning, which involves identifying the needs, resources required, and drivers and barriers to implementation. The term *instructional design* is often used interchangeably with *curriculum development*, but some reserve it specifically to that element of curriculum development that relates to the learning experience, including the delivery and learning strategies.^11^ This is also referred to as *learning design*.

Numerous curriculum development frameworks exist, many of which are derived from the Analysis, Design, Development, Implementation, Evaluation model.^12,13^ Pallium Canada, a non-profit organization established in 2000 to advance primary-level palliative care nationally, has adapted a similar curriculum development approach for its Learning Essential Approaches to Palliative Care (LEAP) program and courses.^14,15^ The framework is used to develop and deploy standardized courses that target different settings and diseases. More than 530 LEAP courses, involving more than 9000 health care professionals from different professions, were delivered across Canada in 2019 alone.

Courses that are intended for large-scale, national deployment necessitate unique learning design considerations. It requires, among others, balancing pedagogical and pragmatic needs. The courses must be resource efficient and able to reach large numbers of learners without diluting the learning experience. This requires intentional design. Given the importance of interprofessional and multidisciplinary collaboration in palliative care, Pallium Canada's LEAP courses are also designed to support interprofessional education (IPE).^16^ This adds additional layers of complexity to learning design.^17,18^

This article describes the LEAP courseware design and the considerations that have informed and underpinned their learning design.^19^ These include options or polarities that present themselves, requiring design decisions that are sometimes informed by evidence, and often by experience, best practices, and the goals and context at hand.

### The LEAP courses

The main goal of the LEAP courses is to provide health care providers across different professions, services, and settings with the core competencies to provide a palliative care approach, also referred to as primary- or generalist-level palliative care.^20,21^ Other goals include to promote interprofessional teamwork; enhance collaboration between services and specialist palliative care teams; and stimulate palliative care-related quality improvements in the health care system.

There are currently 17 different versions of the course, targeting different care settings (community and home care, hospital, long-term care [LTC]), services (such as paramedic services, pediatrics), or disease (such as palliative care in advanced heart, lung, kidney, and liver disease).^15^ The courseware is also being used in some undergraduate and postgraduate medical and nursing curricula. More than 530 course sessions were delivered in 2019 alone to more than 9000 health care professionals of different professions across Canada.

Although there is an overlap across the course versions in terms of design, learning methods, and messaging, each is designed to address the context, competencies, and needs related to that specific setting or disease group. This is achieved by showcasing studies and cases that are germane to those settings and diseases.

The LEAP courses have, until the COVID-19 pandemic, been mainly face-to-face classroom events that are delivered over one or two days. Courses are modular, with each course made up of 7 to 15 modules. The modules cover topics such as the early identification of patients with palliative care needs, self-awareness, decision making, pain and symptom management, essential conversations such as advance care planning and goals-of-care discussions, and addressing psychosocial and spiritual needs. Online learning versions are also available.

The LEAP courseware includes slide-decks with facilitator notes and learning exercises, participant and facilitator manuals, and educational videos. All courses and participants are registered through Pallium Canada's online learning management system (LMS). Facilitators and participants download their respective course materials from the LMS, and learners complete pre- and post-course knowledge, attitudes, comfort, and evaluation surveys online.

## Design Considerations

### Learning theories and overall approaches

The LEAP courses use various design approaches and learning methods to create an interactive, interprofessional, and collaborative learning experience. The designs are influenced by several adult learning approaches and learning theories, including social constructivism, the cognitive flexibility theory, and collaborative learning.^22^ These are applied to the classroom, as well as to hybrid, flipped, and fully online renditions of the courses. While applying them though, their limitations also need to be recognized.^8^

The central premise of social constructivism is that knowledge is a human construction in which learners and facilitators are active participants and not just a passive receptacle.^23^ They bring, in the case of the LEAP courses, pre-existing experiences and insights. The course is designed to encourage learners and facilitators to share some of these experiences and insights. Discussions that provide learning are then centered around these experiences and the cases provided in the courses. Knowledge is constructed as learners make sense of their experiences and learn from each other and the facilitators. Situations that challenge their previous thinking (cognitive dissonance) serve as strong stimuli for new learning. Social constructivism lends itself well to IPE.^24^ The Cognitive Flexibility Theory, which has constructivist links, relates to learning in complex and ill-structured domains, which is very relevant to palliative and end of life care.^25^ The theory pertains to the transfer of knowledge and skills beyond their initial learning situation. In palliative care, for example, it is not unusual that two different patients with very similar diagnoses and burden of disease may require different approaches and treatments because of many different factors. One solution does not necessarily serve as a solution for all patients. The course, therefore, uses many case studies and multiple variations on some cases to illustrate this.

In the LEAP courses, the term “facilitator” is preferred over “teacher” or “presenter,” and “participants” over “learners” to emphasize the interactivity, collaboration, and reflective intent of the courses.

### Design polarities

Additional factors have influenced learning design. These have included factors such as practicality and scalability, cost, resource requirements, and some political considerations. Often, the design has required choices between two opposing options, or polarities, each with their own merits and limitations (Table 1). In this section, we focus on the factors that relate to the classroom course versions.

**Table 1.** Curriculum Considerations and Options in the Design of Pallium Canada's Learning Essential Approaches to Palliative Care (LEAP) Courses

Consideration	Design options		
Target profession	Include all professions	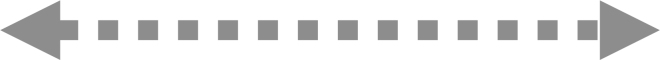	Target specific professions
Profession-specific breakout streams	Incorporate several streams to cater for different professions	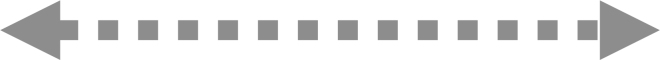	Rely on a single stream that has professions learning together
Course length	Courses of three to five days	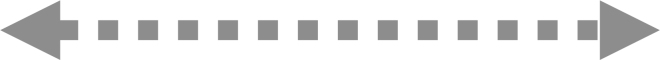	Short courses of one or two days
Content volume	Include all relevant content	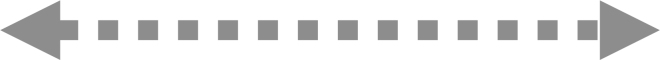	Limit content to only key concepts
Class size	Large numbers of learners per class	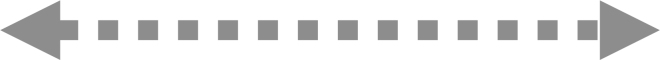	Small class sizes
Integration of topics	Topics are integrated across all modules	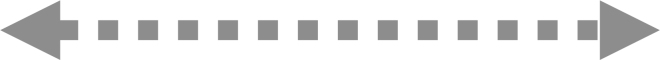	Topics are addressed separately in different modules
Learning methods	Use high-resource, high-impact methods	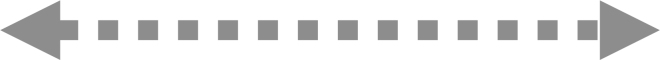	Rely on low-resource, lower-impact options
Classroom or online learning	Only in-person classroom learning	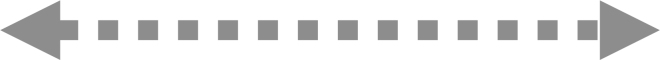	All virtual, online learning
Courseware flexibility	All slides unlocked to allow modifications by facilitators	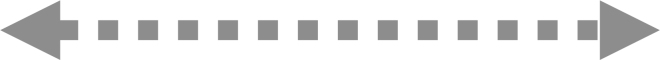	Lock courseware to preclude changes
Regional adaptations	Allow provincial and regional adaptations	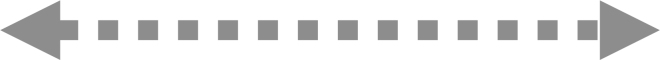	Provide a single generic version
Facilitator criteria	Fully inclusive approach with limited restrictions on who may facilitate courses	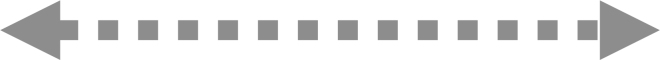	Tight criteria on who may facilitate courses
Pre- and post-course reflection instruments	Pre- and post-course reflection quiz and surveys with multiple items	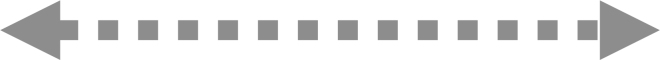	Reflection instruments with reduced items and simplified formats

Each consideration is accompanied by a spectrum of options, with the polarities of these options shown as anchors.

#### Target learners, career stage, level, and competencies

Two of the first design decisions relate to the targeted career stage and level of palliative care. Studies demonstrate learning gaps related to palliative care across the career continuum, from undergraduate and postgraduate training to early or late career practice.^3,26,27^ However, learning needs, motivations, and approaches often evolve and change across one's career.^28^ Courses that rely on learners drawing from their clinical experiences and encounters, such as occurs when constructivism is applied extensively, may be less effective in the case of undergraduate learners who have had more limited clinical exposure compared with seasoned clinicians. Problem-based learning (PBL) or case-based learning (CBL) is applicable across all stages, but the cases that are used, how they are presented and the accompanying questions and depth to which they are explored may vary.

A decision was made to design LEAP courses primarily for postgraduate learners and professionals in practice. However, over the years we have observed that several of the learning methods, including theory bursts, interactive overviews and videos (Table 2), and even the cases, can also be used effectively in undergraduate learning. They require, however, that facilitators or teachers adjust the focus and approach. In undergraduate settings, for example, there is less emphasis on asking learners to share their clinical experiences and insights, and more on using the cases as illustrative examples. Several undergraduate medical, nursing, and health science programs currently use the LEAP courseware in their curricula.

**Table 2.** Learning Methods Used in the Learning Essential Approaches to Palliative Care (LEAP) Courses

Method	Description
Pre- and post-course assessment instruments (surveys and quizzes)	Participants complete a knowledge quiz and attitudes and self-perceived comfort surveys pre- and post-course. Although these are primarily learning tools that prompt reflection, they are also used to evaluate the impact of the courses. Facilitators are provided with the results of the pre-course surveys to allow them to highlight certain areas. Post-course, learners also complete a CTC statement in which they commit to changing three to four things in their practice.^48^ Four months after the course, the Pallium Portal sends them personalized reminders, with their specific commitments, and asks them to reflect on the extent to which they implemented their commitments and provide examples
Case-based learning (long and short cases)	Long and short cases are used. Cases are based on real-life situations and are done by using small or large group learning. Long cases contain several scenes that mimic a real case that unfolds over time. Each scene is accompanied by questions to guide discussions. Short cases (vignettes) consist of only one or two scenes
Lectures (“Theory Bursts” and “Interactive Overviews”)	Serve to introduce key concepts, develop common ground across professions, and challenge preconceived ideas.^49^ Range from 20 to 45 minutes in length. Referred to as “theory bursts” when short and more didactic, and as “interactive overviews” when they include learner engagement methods such as short reflective videos, rapid discussion questions, and polls. They are often bookended by one or more cases
Reflective exercises	Reflective exercises are embedded across the course to prompt reflection on attitudes toward a palliative care approach. Short trigger videos, quotes, reflective questions, and quizzes are used to trigger reflection. These draw on the transformative learning theory, which postulates that transformative experiences, often emotional, can be powerful triggers for learning
Trigger videos (snippets and communication videos)	Short videos (one- to four-minute long) are used to trigger discussions and reflections and for learning communication approaches.^50,51^ Snippets are animated videos that highlight concepts or challenge preconceived ideas. Communication videos show clinicians engaging in various palliative care and end-of-life care discussions with patients and families. They are deliberately scripted to show a mixture of good, borderline, and bad approaches in the same scenario, hence their title “NQR” approach
Small group learning	Learners are divided into groups of not more than 10 learners. A trained facilitator facilitates the discussions. Discussions are centered largely around cases, videos, or reflective questions. Various strategies are used to promote learning engagement.^52^
Large group learning	Some issues, videos, and cases are discussed as a large group, mainly for time efficiency or if the discussion benefits from many perspectives. However, because LEAP courses limit the number of learners to no more than 30 learners, interactivity is still retained.
Role play	Role play is used in the communication modules to allow learners to learn and practice communication skills. In some cases, group role play is used in which two volunteers play the parts of health care professionals and patients or families, but facilitators get the larger group to provide prompts to the “professional”
White board	Facilitators are encouraged to use the whiteboard to illustrate some points (e.g., show an opioid switch calculation)
Clinical parking lot	Use a white board or flip chart on which to list questions or issues that arise that cannot be addressed immediately but are listed to come back to at a later stage
QI parking lot and module	Education alone may not change behavior, requiring additional strategies. QI may enhance the impact of education interventions.^39^ Participants invariably identify, during the course, things that could be improved in their respective services. At the end of the course, in the “Effecting Change” module, facilitators return to these ideas and encourage participants to activate QI activities around some of these

CTC, commitment to change; LEAP, Learning Essential Approaches to Palliative Care; NQR, Not Quite Right; QI, quality improvement.

A parallel design decision relates to the competencies to be addressed. Given Pallium Canada's mission to advance the palliative care approach, this has required distinguishing between generalist-level competencies and specialist-level palliative care competencies, which at times can be challenging.^29^ In the early years (2000–2012), Pallium Canada relied on a combination of methods to establish the targeted competencies. It did this through a combination of literature reviews and input from targeted learners and palliative care experts. We found the Developing a Curriculum approach particularly useful in that it brings together, through focus groups, professionals who are well acquainted with the roles and maps out what their work realities are and the knowledge, skills, and attitudes that they need to perform effectively.^30^ More recently, Pallium Canada has used competencies elucidated by various palliative care societies and associations.^31^

Lastly, although the LEAP course designs are not modified based on whether learners have enrolled voluntarily or because of obligatory course or work requirements, we have observed that facilitators sometimes need to alter the focus of the discussions, particularly in the introductory modules of the courses. More time and focus may be spent on covering the course materials that highlight the need for palliative care, including studies, when learners' appear more skeptical. This, however, is purely an observation and whether or not differences truly exist between these groups warrants further study.

#### Target professions and IPE

Targeting a single profession, such as physicians or nurses, simplifies the design process as one needs to address the competencies and scope of practice of only one profession. Targeting all professions is most inclusive but also presents major challenges. Some “palliative care approach” competencies overlap across professions, whereas others diverge in scope and depth. We have found that the most feasible and practical has been to target physicians and nurses as the primary audience, while providing pharmacists, social workers, and allied health professionals an opportunity to participate as well. Strategies such as prompts to solicit input from other professions are incorporated to support learning for other professions. Although the courses are designed as interprofessional workshops, they may also be delivered as “uni-professional” events when opportunities arise where only one profession is assembled, such as profession-specific conferences or services.

The integration of interprofessional learning has been guided by evidence and best practices in IPE.^17,18,32^ The design teams for each of the LEAP course versions, for example, are interprofessional to ensure the inclusion of the various perspectives. The courses promote appreciation of each profession's contributions and realities, and the creation of shared understandings.^33^ The CBL can support IPE, especially when cases are constructed to elicit different perspectives and multi-professional input.^32^ Moreover, interprofessional collaboration is role modeled when course facilitation teams are interprofessional. Course facilitators are required to draw out different professions' perspectives during the learning experience.

Group balance is recommended in IPE.^17^ However, this is often not possible because the learners who attend an LEAP course are usually proportionately representative of the workforce in that setting. The LEAP Hospital courses, for example, usually have large numbers of nurses compared with physicians or pharmacists.

Facilitators play a key role in creating a learning environment that is conducive to IPE.^17,34^ Among others, facilitators must be sensitive to the dynamics of IPE. This includes being alert to interprofessional learning moments, in addition to the formal prompts embedded in the cases. This occurs, for example, when a learner raises a question or shares an experience that a facilitator could then use to highlight the contributions and roles of different professions. Learners need to be made to feel like equals and free to share their profession's perspectives. Facilitators must be ready to encounter and manage interprofessional friction and address issues of power and hierarchy that may arise. “Turf” or domain protectionism needs to be addressed when one profession may claim exclusive ownership to a particular domain, such as goals-of-care discussions, psychosocial care, or pharmacological management. A social worker may, for example, suggest that only they are able to address the social needs of a patient or a physician state or implies that goals-of-care discussions are exclusively their responsibility. Clearly, all team members should be able to identify social needs, leaving complex social needs to be addressed by the team's social workers. Similarly, goals-of-care discussions can also, to varying levels, be undertaken or prompted by other professions while respecting their scopes of practice.

#### Profession-specific breakout streams

To address profession-specific competencies and learning needs, breakout parallel sessions in each course for different professions (or clusters of professions) can be used. However, this may require extra facilitators and venues with additional break-out space, making it cost- and resource-prohibitive in some cases. However, facilitators are granted latitude to cluster professions during the small group break-out learning sessions when there is a sufficiently large group of learners from a particular profession or profession cluster such as allied health professionals to make it feasible. In the course version that target LTC settings (LEAP LTC), there are mandatory break-out sessions for personal support workers (PSWs), as they constitute a large care-provider group with unique skills sets in this setting.

#### Class sizes

Class size presents a tension between ideal adult learning environments and pragmatism. From a learning perspective, the number of learners affects interactivity; the smaller the number, the more opportunities for interactions between learners and faculty.^35,36^ However, too small a number hinders large-scale deployment because only a small number of professionals can be trained in any given session. We have found that, for the LEAP classroom course versions, a maximum of 30 participants and a ratio of 1 facilitator per 10 learners offers the best balance between these polarities.

#### Course length and content volume

The scope and depth to which content is covered in LEAP courses and included in facilitator manuals is an ongoing challenge. Excessive volume results in information overload and a pressured learning experience for participants and facilitators alike. Too little, on the other hand, results in suboptimal coverage of topics and inadequate support for some facilitators, especially those who do not have ready access to journals and other literature. The courseware helps them stay in touch with evolving evidence and best practices.

Lengthening the courses by a day or two has been trialed. The downside is that it creates a time barrier that deters larger numbers of health care professionals from attending courses or participating in all the modules. Services must find replacement staff (backfill) and pay the salaries of both course attendees and backfill staff. One- or two-day offerings and hybrid programs (with online and classroom learning) appear to provide a good balance that achieves optimal workforce outreach.

#### Horizontal or clustered integration of topics and learning objectives

In real life, patients often present with several needs at any given time, and with varying physical, psychological, social, or spiritual needs across their illness journeys. Courses can reflect this by using scaffolded cases that unfold across the entire course, with multiple scenes within and across modules. Each new scene is accompanied by questions, reflections, or exercises.

Trial and error have shown us that it is more practical to scaffold cases within modules instead of across modules. Clustering topics helps organize the courses and allows for modules to be used alone if needed. The cases do, however, introduce some domains outside of the module's specific focus. In the pain management module, for example, the patient in the case study also experiences social and psychological needs. Some competencies, such as decision making and ethics, are addressed horizontally across all the modules.

#### Courseware flexibility

The LEAP courseware is locked to prevent changes being made without Pallium Canada's permission. Although unlocking would allow facilitators to “personalize” the courseware, experience has shown that this ultimately compromises quality and credibility as materials are modified without peer review or quality control. To circumvent this and provide some flexibility, LEAP facilitators may showcase local resources such as clinical guidelines to some by toggling in and out of an LEAP slide deck.

The preferred mode of delivery is to have the courses delivered in single sessions, with all modules delivered back-to-back. This appears to enhance group cohesion and course continuity. However, the design allows the courses to be split into several sessions if necessary, to accommodate service needs and requirements. The two-day courses, for example, can be split into 2 one-day sessions, or 4 half-day sessions. Modules may also be delivered separately as self-standing learning events, such as occurs in academic detailing. This is particularly useful to undergraduate and postgraduate educators who can insert select modules into existing curricula.

#### Regional adaptations and language versions

The LEAP courses are delivered across Canada's 13 provinces and territories. There are variations across jurisdictions in terms of advance care planning laws and terminology, drug coverage, and the availability of palliative care services. Developing different course versions to accommodate these regional differences is challenging. The courses are therefore generic, but facilitators are prompted to highlight regional variations for their jurisdictions. Most courses are also adapted into French.

The LEAP courseware has undergone limited piloting outside of Canada. These included translations and adaptations for use in a medical undergraduate course in Spain, and a course for community- and hospital-based physicians and nurses in Portugal. Adaptations were also tested, in English in the Caribbean and in a medical school in New Zealand. The courses needed little redesign and the content modification related to local contexts and realities, mainly around availability of medications locally, local regulations in areas such as advance care planning, and local cultural realities such as prognostic disclosures and scopes of practice across professions.

#### Facilitator criteria, professions, and certification

Effective facilitation is critical to ensure an optimal constructivist learning experience. Polar options present themselves in terms of facilitator criteria. A “closed” approach would rely on a relatively small select group of highly skilled facilitators to deliver all the courses. This would, however, not be practical and would impede large-scale deployment. It would also not promote rapport-building between participants and local palliative care providers if the program relies on “flying” in certified facilitators from other regions. A completely open model, on the other hand, in which anyone can present the curricula regardless of clinical or teaching and facilitation expertise, could compromise the quality of the learning experience.

Facilitators are, therefore, required to be experienced palliative care professionals; most are physicians, nurses, and social workers with advanced training and certification in palliative care or with advanced clinical experience providing specialist-level palliative care. Current or recent clinical experience provides authenticity and centers learning on practical approaches. Facilitators can only facilitate a course once they have completed a one-day course called LEAP Facilitator, and have successfully co-facilitated one to two courses with an experienced facilitator. The LEAP Facilitator orientates educators to the design content of LEAP courses, their goals; provides hands-on skill training on how to ensure a highly interactive, learner-centered, learning experience; and also supports IPE. More recently, additional competencies have been delineated for online facilitation and a new facilitator course has been added to train online facilitators. There are currently more than 900 certified facilitators across the country to deliver classroom-based courses, and more than 100 trained to deliver webinars for the online course versions. The large pool of facilitators, with their presence across all Canadian jurisdictions, enhances access to a facilitator even if an organization or service hosting a course does not have in-house palliative care clinicians.

### Learning methods

The LEAP courses incorporate various learning methods (Table 2). Although these are largely selected based on the pertinent learning objectives, other factors must also be considered. These include feasibility and resource availability for large-scale deployment. Balancing these factors sometimes also calls for design compromises. For example, although simulated patients are effective methods to learn communication skills, they are very resource-intensive and not practical for a short course that has to cover other competencies.^37^ The LEAP courses, therefore, largely use education videos and role play instead.

The CBL is used instead of PBL. This leverages CBL's efficiency as a more structured guided inquiry method for short courses.^38^ Cases are discussed in small or large groups. For small group learning, some suggest group sizes of 10 or less.^39^ Others posit that the number of learners should not conform to any set rule but depends on the goals and objectives of the program, and the experience of the facilitators.^39^ Short lectures, in the form of overviews, are also used for their efficiency to introduce key concepts and develop common understandings across professions. They are often book-ended by cases to provide clinical contexts and application.

### LEAP adaptations for virtual learning

More recently, in response to the COVID-19 pandemic, the LEAP courses have transitioned to being fully online. Previously, some course versions had used blended delivery approaches with classroom and online components.^40^ In LEAP Facilitator, for example, a hybrid approach was used in which self-learning online modules replaced portions of the classroom course, whereas the classroom component focused on experiential learning. Pallium had also developed a suite of 15 interactive self-learning online modules, each 10- to 30-minutes long, to complement classroom learning in a flipped learning model. In a flipped learning model, learners usually undertake self-study, followed by classroom learning. The current fully online LEAP versions use a similar approach, but the classroom in-person component is replaced with four interprofessional live webinars, each 90 minutes long. The webinars use CBL to provide clinical context and application to what was learned in the self-learning modules.

Webinar class sizes have also been limited to a maximum of 30 participants to ensure interactivity. Break-out sessions are also incorporated. However, because the pool of trained online facilitators was relatively limited at the start of the pandemic, breakout sessions were used more sparingly than in classroom in-person events. Once a larger pool of facilitators was trained to teach online, more breakout sessions were incorporated. This switch to virtual learning has been successful in that more than 150 fully online LEAP courses were delivered from April 2020 to March 2021, with almost 3000 learners and very positive ratings by learners on the learning experience.

## Course Evaluations and Impact

Ultimately, the proof of whether the design is successful lies in the program and course evaluations and studies of impact across various levels and domains. A large study was undertaken of all learners who participated in LEAP courses from April 2015 to March 2017. Table 3 summarizes the responses to two of the seven course evaluation questions by 3045 out of 4636 learners who participated in LEAP Core sessions (response rate 65.7%). The two questions are used by Pallium Canada as global indicators of course success. Relevancy has recently been highlighted as an important indicator.^41^ The large majority of participants, across professions, rated the course as relevant to their work and expressed that they would recommend it to colleagues. Qualitative analyses revealed interactivity, IPE, the use of narratives and cases, and the quality of facilitation as course strengths. Areas for improvement included reducing course content. Analyses of the four-month post-course commitment-to-change reflections noted that on average 65% to 75% of commitments post-course were being implemented with examples of benefits to patient care and the health care system provided.^42^ Significant pre- versus post-course improvements in knowledge, attitudes, and comfort levels related to providing a palliative care approach were noted across professions.^43^ These results are reassuring from a design perspective. A full description of all the results will be published elsewhere. Similar results were found with the LEAP LTC courses.

**Table 3.** “Proportion of Learning Essential Approaches to Palliative Care (LEAP) Core Course Participants, Overall and by Profession, Who Responded “Strongly Agree” or “Agree” to Two Evaluation Questions Related to the Learning Experience.” (For all LEAP Core version courses delivered from 1 April 2015 to 30 March 2017)

	Profession	Total number of learners (%)^a^	Number (%) of responses	Participants (%) who responded “Strongly Agree” or “Agree”^b^
“The course was relevant to my practice”	Physicians	878 (18.9)	662 (75.4)	640 (96.7)
	Nurses	2990 (64.5)	1973 (66)	1919 (97.3)
	Pharmacists	100 (2.2)	74 (74)	65 (87.8)
	Social workers	127 (2.7)	80 (63)	63 (78.8)
	Others	541 (11.7)	256 (47.3)	231 (90.2)
	Total	4636 (100)	3045 (65.7)	2918 (95.8)
“I would recommend the course to colleagues”	Physicians	See above	See above	631 (95.3)
	Nurses	See above	See above	1934 (98)
	Pharmacists	See above	See above	70 (94.6)
	Social workers	See above	See above	71 (88.8)
	Others	See above	See above	243 (94.9)
	Total	See above		2949 (96.8)

^a^ Percentage refers to the proportion that profession was represented relative to all learners.

^b^ The denominator is the total number of responses to the survey received from that profession.

Evidence of impact has been found in other studies. Evaluations of the INTEGRATE Project, a multipronged intervention that included training of staff at cancer center programs and family health clinics with LEAP courses, found improved earlier identification of patients with palliative care needs, increased use of palliative care services, and improved professionals' skills.^44–46^ In an evaluation of the “Paramedics Palliative Care” project in two provinces, in which LEAP Paramedic training was applied alongside policy and procedure changes, patients and families reported high degrees of satisfaction, particularly being able to be cared for at home.^47^ Paramedics reported increased comfort, confidence, and joy providing palliative care, whereas patients and families reported better symptom control, quality of life, and gratitude for being cared for in their homes.

## Future Design Directions

Online learning, including flipped and hybrid options, provides a potential solution to addressing the design challenge related to addressing profession-specific learning needs in IPE. We have started to develop profession-specific self-learning modules to complement the other course components. Moreover, content that introduces learners to contributions by other professions can also be introduced and then highlighted in the live webinars. Increasingly, nursing aides are also being included in LEAP training, particularly as these providers play key roles in providing care in the home and LTC (nursing homes) settings in Canada. A new LEAP PSW course has just been launched. In the interim, it is a series of online self-learning modules. The plan is to add live webinars in the future, and to have them later also participate in interprofessional classroom or live webinar sessions alongside other professions. The premise is that they will feel more confident to participate alongside other professions once they are empowered with some basic knowledge and understanding of the field.

Work is also underway to develop some self-standing modules that provide updates on the current management of various non-cancer illnesses. The LEAP facilitators, although experienced palliative care clinicians, often report feeling a need to update their own knowledge in these areas, especially when they are asked to facilitate a disease-specific course such as LEAP Renal, LEAP Heart, and LEAP Lung, to nephrologists, cardiologists, and respirologists.

Finally, future work will include repackaging the videos to allow them to be used as interactive self-learning online modules. Additional videos that reflect a variety of care settings also need to be developed to better reflect realities in non-cancer clinics such as dialysis units (for LEAP Renal) or emergency departments (for LEAP ED).

## Funding, Distribution, and Spread Strategies

Although a detailed description of the program's spread and scale-up strategies is outside the scope of this article, two key approaches merit attention. First, although courses are developed and maintained centrally by Pallium Canada, local partners such as palliative care services, universities, home care agencies, hospitals, and nursing homes organize and deliver the courses. They draw on local certified facilitators to present the courses.

Second, a social enterprise model has evolved to ensure program sustainability. Government funding has been critical to support early curriculum development and testing, implementing an LMS, and creating a large community of practice of curriculum developers and facilitators across the country. However, this funding source has been precarious and, for some years, absent. Pallium Canada, therefore, relies now on other funding sources, primarily revenues from course registrations and philanthropic contributions. The costing model is based on the principles that the registration fee should be fair and acceptable to learners and organizations that may subsidize them, support Pallium's ongoing operations and continuous development and research activities, and allow local organizers to cover the costs of organizing and delivering a course. The latter include honoraria for facilitators and the costs of providing a venue and meals. In Canada, the fee for a two-day medical education event is generally about $600 to $800, on average. Local organizers may charge similar rates (which vary from province to province, profession, and course type) for an LEAP course and Pallium retains a quarter to a third of that. Similar fees are charged for facilitator training.

A fine balance is needed between offering courses for free and excessive registration fees. The former would render the program unsustainable, and we have also observed high levels of no-shows (up to 30% of registrations in some cases). High fees, on the other hand, would pose significant barriers to adopting the curricula and participating in the courses.

## Conclusion

Designing palliative care interprofessional CPD courses for large-scale, national deployment requires making a number of decisions that impact the learning experience. Several design options present themselves, requiring curriculum developers to make choices or compromises between various options. Incorporating IPE adds further complexity to designing the learning experience.

Although there is a considerable palliative care literature that describes curricula, learner reactions to them, and their impact on aspects of competency such as knowledge, attitudes, and self-perceived efficacy, a few publications specifically explore the design considerations and polarities that underlie them, especially with respect to postgraduate IPE. Educators are often left to learn by trial-and-error. Some are therefore calling for greater clarity and exploration of the design of continuing IPE interventions, in addition to evaluating their impact.^18^

Making design decisions should ideally be informed by evidence, but often educators need to rely on best practices, experience, and ongoing program evaluations. In this article, we have explicitly explored the design considerations and decisions that underlie interprofessional palliative care courseware intended for national deployment. There is a need for ongoing research into the impact of different design choices, including learning methods, alone or in combination. This also extends to different delivery methods such as classroom, flipped, blended, and fully virtual learning, allowing us to harness their respective strengths and avoid their pitfalls.

## Authors' Contributions

J.P.: Co-founder of Pallium Canada, conception and design, lead for curriculum development and curriculum evaluation, and drafted manuscripts. G.G.: LEAP course design and curriculum lead, manuscript input. R.S.: LEAP course design (online) and curriculum lead, LEAP facilitator, and manuscript preparation. I.H.: LEAP course design and curriculum lead, manuscript input. E.A.: LEAP course design, curriculum lead, LEAP facilitator, and manuscript preparation. J.F.: Operations supporting the courseware development, manuscript preparation.

## Acknowledgments

The LEAP Course designs, facilitation, and deployment have been made possible by many people. The authors wish to thank the many curriculum team leads and members of the various LEAP courses. The team leads include Lori Teeple (LEAP Core and LEAP Facilitators), Jill Marcella (LEAP LTC), Mary-Lou Kelly, PhD (LTC), Dr. Ingrid Harle (LEAP Hospital), Daphna Grossman (LEAP Hospital), Erin O'Connor (LEAP ED), Dr. Lisa Fisher (LEAP ED), Dr. Yoko Tarumi (LEAP Onco), Dr. David Henderson (LEAP FT), Dr. Denise Marshall (LEAP Core), Dr. Vanita Jassal (LEAP Renal), and Dr. Valerie Schultz (LEAP Renal). The authors would also like to thank the staff at Pallium Canada who have supported the development of the LEAP courses in the past and currently (Nathalie Gravelle-Wray, Odete Carreira, Gaelle Parsons, Diana Vincze, Brady Riordan), Michael Aherne who contributed to the early course designs and concepts, and Pallium Canada leadership over the years, including Michael Aherne, Kathryn Downer, EdD, Jeff Moat, Jonathan Faulkner, and Srini Chary. Thank are due to Lamia Hayawi and Tammy Tsang who helped prepare the article.

## Ethical Approval and Patient Consent

This article describes the learning design decisions that underpin Pallium Canada's interprofessional LEAP courses. It is not a research study and therefore, neither ethical approval nor patient or participant consent was required.

## Abbreviations Used

CBL: case-based learning
CPD: continuing professional development
CTC: commitment to change
ED: emergency department
IPE: interprofessional education
LEAP: Learning Essential Approaches to Palliative Care
LMS: learning management system
LTC: long-term care
NQR: Not Quite Right
PBL: problem-based learning
PSW: personal support worker
QI: quality improvement
